# Follow-Up Weekly Training Distribution and Accumulated Internal Load Effects on Young Football Players’ Well-Being, Physical Fitness, and Technical Performance

**DOI:** 10.3390/sports12010023

**Published:** 2024-01-09

**Authors:** Yutthana Riansoi, Nuntapol Tongnillpant, Sakdarin Thammawong, João Ribeiro, Rodrigo Zacca, Phornpot Chainok

**Affiliations:** 1 Faculty of Science, Chandrakasem Rajabhat University, Bangkok 10900, Thailand; yutthana.r@chandra.ac.th; 2 Sports Science Centre, Sports Authority of Thailand, Bangkok 10900, Thailand; bombswu@yahoo.com; 3 Faculty of Sport Science and Health, Thailand National Sports University, Udonthani Campus, Udonthani 12110, Thailand; thsakdarin@gmail.com; 4 Center of Research, Education, Innovation and Intervention in Sports (CIFI2D), Faculty of Sports, University of Porto, 4099-002 Porto, Portugal; joaoribeiro@fade.up.pt; 5 Research Center in Physical Activity, Health and Leisure (CIAFEL), Faculty of Sports, University of Porto (FADEUP), 4099-002 Porto, Portugal; rzacca@fade.up.pt; 6 Laboratory for Integrative and Translational Research in Population Health (ITR), 4050-600 Porto, Portugal; 7 Faculty of Sports Science, Burapha University, Chonburi 20131, Thailand

**Keywords:** soccer, pre-season, internal training load, wellness status, follow-up

## Abstract

This study aimed to quantify the relationships among internal training load, wellness, physiological, isokinetic profile, and technical performance and assessed changes before and after a six-week pre-season structured combining physical fitness, small-sided-game and skill-based training program in young soccer players. Forty-five national-level young male soccer players (16.8 ± 0.9 years) were evaluated. There were no significant correlations between the wellness index and the physiological, isokinetic, or specific soccer skill parameters. Moderate correlations were observed between fatigue and stress (r = 0.47, moderate, *p* < 0.01), fatigue and average muscle soreness (r = 0.38, moderate, *p* = 0.01), and a large correlation between average stress and muscle soreness (r = 0.53, large, *p* < 0.01) were presented. All physiological, isokinetic, and technical parameters improved after a six-week pre-season training program (0.1 to −61.0%). Significant alterations in the physiological domain were observed after a six-week period, particularly in the fatigue index of the Bangsbo sprint test, YO-YO IR1 performance, and YO-YO peak La^-^ (*p* < 0.01; −61.0%, 25.3%, and −19.8%, respectively). As such, the implementation of structured training and the monitoring of stress responses can lead to appropriate adaptation and balancing in the psychological and physical well-being of young national-level soccer players, particularly in the pre-season period.

## 1. Introduction

Soccer (or football) is characterized by intermittent efforts at different intensities and durations in which its performance is determined by the integration and interaction of physiological and biomechanical aspects, tactical-technical ability, external-internal training load optimization, and mental skills, among others [1,2,3]. However, in light of recent trends in young soccer performance, there is growing concern about detailed information on the relationship of comprehensive factors to young soccer performance [2,4,5]. Thus, a deeper understanding is possible when exploring training program issues and integrated longitudinal data assessment in parallel [2,3].

When designing pre-season soccer training, it is important to incorporate various components of physical fitness-based (FBT) training, including anaerobic and aerobic fitness, power, agility, speed, and strength training, plyometric exercises, short sprints, and eccentric hamstring exercises [6,7,8,9]. Additionally, small-sided and conditioned games (SSG) should be included, as well as skill-based training (SBT), to encourage physical, physiological, and technical improvements [6,10]. This study of short-term pre-season training, which includes physical fitness, psychological factors, technical skills, and team tactics, significantly influences the development of jumping and running ability, speed and strength-based conditioning, motor skills, and aerobic endurance in young soccer players [5,10].

Despite this interest in pre-season training, different approaches have been identified to address the challenges coaches face in managing the amount of internal and external training load and their well-being status (such as stress, fatigue, muscle soreness, and sleep quality) during the pre-season period. These approaches aim to monitor the improvement of physical and physiological performance [1,2,6], account for variations in training responses [11], and prevent neuromuscular fatigue and soreness, which can increase the risk of injuries and negatively impact skill and balance in soccer players at all levels, from young national players to elite international players [5,12]. From these perspectives, subjective and objective measurement and monitoring of the complex process of the individual stress response to training, particularly in young Soccer players, is necessary to maintain the unique balance required for homeostasis [13]. Monitoring training load and wellness status can be a reliable, responsive, and effective indication of training load and enhancing performance [13,14].

Studies based on the investigation of interactions and associations among training quality, well-being status, physical fitness, and soccer performance in the season have been extensively utilized and well-described for elite professional soccer players [15,16]. However, in light of recent monitoring of the complex adaptation process and subsequent performance in young soccer players, there is now considerable concern about comprehensive subjective measures that contribute to and are related to training periodization, particularly prior to the pre-season, and overall performance has not been completely elucidated. In this sense, comprehensive subjective information to individually prescribe and follow a weekly training program for soccer players is important for sports performance development, especially during pre-season training.

The aims of this study were (i) to explore the relationships among internal training load and wellness, physiological, isokinetic profiling, and technical performance, and (ii) to evaluate changes and improvements in soccer-specific fitness, isokinetic profiling, and specific skills before and after a six-week pre-season structured training program combining physical fitness, small-sided games, and skill-based training programs in young national-level soccer players. Based on the relevant literature [15,16,17], we hypothesized that the accumulated internal training load might partially relate to wellness status, physiological, isokinetic profiling, and technical performance and also explain the variation of youth players’ fitness levels during the pre-season.

## 2. Materials and Methods

### 2.1. Participant

An a priori power analysis to determine sample size was completed using G*Power 3.1.9.2 software. The Type I alpha (error level) at 5% and a Type II beta (error level) of 5% (or a power of 95%) were set a priori. This analysis showed that for a medium effect size of 0.50, an error probability of 0.05, and a power of 0.95, the sample size would need to be n = 45. Forty-five high-level youth national soccer players from the same elite soccer team (excluding goalkeepers; age: 16.8 ± 0.9 years old; body height: 1.7 ± 0.1 m; body weight: 63.3 ± 7.1 kg; body mass index: 21.6 ± 2.3 kg·m^−2^). The longitudinal single cohort study was conducted and tested young national-level soccer players before and after six weeks of preseason training to examine the relationships between internal training load and wellness, physiological, isokinetic profiling, and technical performance. We also evaluated the magnitude of changes in physiological fitness, isokinetic profiling, and specific soccer skills. The first experimental testing (PRE) took place immediately in the first week, when the players had just resumed their training after four weeks of no systematic work during the off-season. The second experimental test (POST) was conducted six weeks later. Young national-level soccer players trained seven sessions a week (~90 min/session) focused primarily on technical skills and general physical fitness. Parents were informed about the benefits and risks of taking part in the current study prior to signing an informed consent form, which was approved by the ethics board of the local university (code nº001/2560) and performed according to the Helsinki Declaration.

### 2.2. Procedure

**Figure 1:**
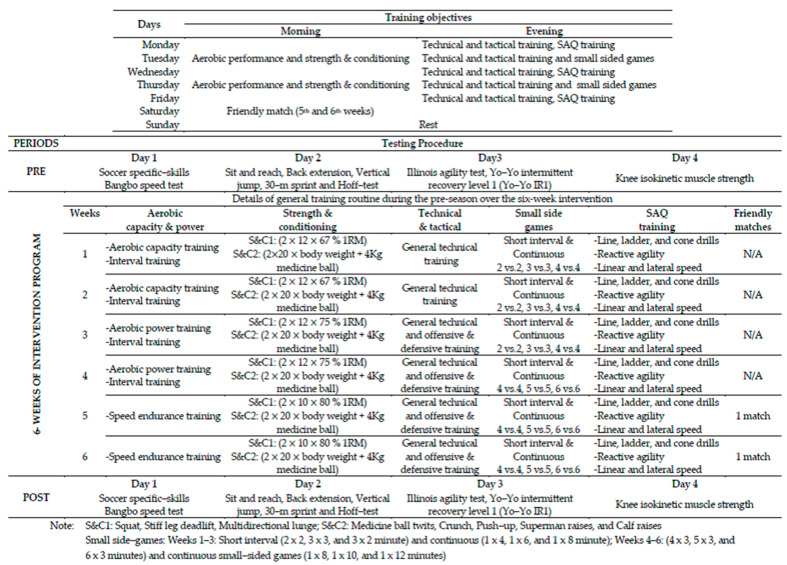
An overview of the six-week pre-season structured combined training program and testing procedure.

In the present study, we selected test items that have been reported as having a direct impact on improving young soccer performance and soccer-specific demands, including physical performance tests (flexibility, agility, power, and speed) [18,19], Soccer-specific fitness tests (YO-YO intermittent recovery test: YO-YO IR1, Hoff test, and Bangsbo sprint test: BST) [20,21], and Soccer-specific skills (slalom dribble, lob pass, short pass, and shooting) [4]. The pre-test was taken four consecutive days before the start of pre-season training: on day one, soccer-specific skills and the Bangsbo speed test were conducted; on day two, sit and reach and back extension, vertical jump, 30 m sprint, and the Hoff-test were conducted; on day three, the Illinois agility test and the YO-YO IR1 test were conducted; and on day four, knee isokinetic muscle strength was carried out. To ensure optimal recovery, post-testing was performed four consecutive days after the last training session, following the same sequence and procedures as pretesting. All physical performance tests, soccer-specific fitness tests, and soccer-specific skills were carried out on a natural grass soccer field during the day between 16:00 and 18:00 to control the circadian rhythm, which can have a direct impact on metabolism and other bodily functions. Players wore soccer clothing during all field tests and shorts and running shoes during evaluation in isokinetic conditions. An overview of the six-week pre-season structured combined training program and testing procedure is presented in Figure 1.

Four physical performance parameters were measured annually (sit and reach, Illinois agility test, counter movement jump (CMJ), and 30 m sprint test). The sit-and-reach test was used to measure the flexibility of the hamstring muscles and lower back with their feet flat against the sit-and-reach testing box (Baseline Sit n’ Reach flexibility box, White Plains, NY, USA). Two trials were given for each test, and the better of the two trials was retained for analysis. CMJ performance was measured using the BTS G-Walk at a frequency of 100 Hz (BTS Bioengineering Company, Rome Italy). The G-Walk wireless sensor device was placed on an elastic belt and worn around the waist of the person being evaluated. The subjects started the test by standing up with their feet shoulder-width apart and their hands on their hips, followed by a downward countermovement followed by an explosive upward movement. Two trials were given, and the highest jump of the two trials was retained for analysis.

The Illinois agility test was used for testing acceleration and deceleration speed, simple reactions, as well as changes of direction. The test consisted of two maximal sprints with a two-minute rest between each sprint. The time was recorded using the kinematic measurement system (KMS) infrared timing light system interfaced with compatible computer software (Fitness Technologies, Adelaide, Australia). The fastest trial was selected for analysis. A 30 m speed test was also measured with a KMS infrared timing light system positioned at 10 m, 20 m, and 30 m from the starting line. To reduce the effect of fatigue on the results, the young soccer players were instructed to sprint at maximum speed and given two attempts for each condition, with one minute of rest in between. The fastest times at the distances were recorded for data analysis.

Three soccer-specific fitness tests were measured annually: the YO-YO IR1 [22], the Hoff-test [20], and the Bangsbo sprint test (BST) [20]. The YO-YO IR1 [22,23] was used to estimate maximal oxygen uptake ($\dot{V}$O_2max_). The test consisted of 2 × 20 m shuttle runs at a progressively increased speed controlled by an audio metronome. The testing was concluded when young soccer players were not able to maintain the required speed twice to reach the finishing line in time [22]. The distance covered at that point was recorded and further considered to estimate $\dot{V}$O_2max_ from the following equation [23]:

$$\dot{V}O_{2\max}\;(\mathrm{mL}\cdot {\mathrm{kg}}^{-K}\cdot \min^{-m})=\mathrm{distance}\;\mathrm{covered}\;(m)\times 0.0084\;(\mathrm{mL}\cdot {\mathrm{kg}}^{-K}\cdot \min^{-m})\times m+36.4\;(\mathrm{mL}\cdot {\mathrm{kg}}^{-K}\cdot \min^{-1})$$

The Hoff test consisted of specific dribbling on a 290 m circuit track to cover the most distance in 10 min [20]. Three young soccer players were tested at the same time and wore T-shirts with the numbers 1–3 in the same order to be easily identified. At 3, 5, 7, and 9 min, each player was informed of the time remaining. The BST, which consisted of seven maximal 34.2 m sprints followed by 25 s of active recovery in each lab, was used to assess the aerobic capacity of young Soccer players. The time was recorded using the KMS infrared timing light system (Fitness Technologies, Adelaide, Australia) at the start and end lines of the test. To control the consistency of recovery time, verbal feedback was given at 15, 20, and 24 s [19]. Performances were measured as the shortest sprint time, mean time, and fatigue index calculated by the following equation:

Fatigue index (%) = (mean time/fastest sprint time·100) − 100.

Capillary blood samples for [La^−^] were collected from an earlobe prior to exercise and immediately after the YO-YO IR1, Hoff test, and the BST at the first, third, fifth, and seventh minutes of recovery using the Portable Lactate Scout^®^ (LS, SensLab GmbH, Leipzig, Germany).

The hamstring-to-quadriceps strength ratio (H/Q ratio), which has been linked to knee function prediction and muscle strength imbalance, was used to investigate bilateral and unilateral strength asymmetries in young Soccer players. The knee flexion and extension torque of the young soccer players were evaluated in isokinetic conditions at a speed of 60 deg·s^−1^ (slow velocity), 180 deg·s^−1^ (medium velocity), through a knee range of motion of 0° (flexed) to 90° (full extension). All isokinetic contractions were performed and carried out separately for both legs using a Biodex System 4-PRO^®^ (Biodex Medical Systems Inc., Shirley, NY, USA) dynamometer to determine the H/Q ratio at each velocity, and all trials were randomized with respect to the speed and leg tested.

Four soccer tests (dribbling slalom, wall pass, long pass, and shooting) were used to assess specific technical skills [4]. The soccer-specific technical skills tests were performed on a soccer field, and players wore soccer clothing and shoes. Participants performed two consecutive trials, with a rest of one minute between trials of the slalom dribble test, followed by a wall pass, lob pass, and shooting accuracy. The dribbling slalom required participants to dribble around nine cones (2 m apart) from start to finish lines (36 m) as quickly as possible. A wall pass test was carried out by passing the ball to a target on a wall (112 cm high and 244 cm wide) located 2 m apart from the starting line. The aim of this test was to accumulate as many passes in 20 s as possible, and the highest number of passes were recorded for data analysis. A test of lob pass requires a young player to kick a ball from 20 m into a zone that is divided into three concentric circles with diameters of 3, 6, and 9 m, with points awarded in the circle where the ball first landed being 3, 2, and 1, respectively. Ten attempts with five feet each were performed for a total of 30 points. In a shooting accuracy test, young soccer players were required to kick the ball from a distance of 20 m at a 16-m-wide goal target divided into five goal targets. The five goal targets included three points of 2 m wide in the center, two points of two areas of 3 m on each side of the center, and one point of two areas of 4 m wide at each extreme. Ten shots were taken, five from each foot, for a total of 30 points.

For internal training load, the rate of perceived exertion (RPE) was measured weekly using the Borg CR-10 scale of self-regulation of intensity [14]. The s-RPE was expressed in arbitrary units (AU) by multiplying the duration (mins) of each training session, and the internal training load was then analyzed from the equation [8]:

Internal training load = session − RPE (AU) × training duration (min)

A self-reported questionnaire with a 7-point scale including perceived levels of stress, fatigue, muscle soreness, and sleep quality was evaluated separately, and the sum of the four subjective ratings was reported as Hooper index scores, which are used to daily evaluate the wellness status of young soccer players [22,24].

### 2.3. Statistical Analysis

Basic exploratory and descriptive statistics were computed using SPSS Statistics for Windows Version 24.0 (IBM Corp., Armonk, NY, USA), aiming to detect potential errors in data entry and eventual outliers, as well as assess data distribution normality (the Shapiro–Wilk test). A paired t-test was used to compare differences between PRE and POST off-season for each variable. Effect sizes (Cohen’s d) were calculated with the following criteria: 0 to 0.19 trivial, 0.2 to 0.59 small, 0.6 to 1.19 moderate, 1.2 to 1.99 large, 2.0 to 3.99 very large, and >4.0 nearly perfect [25]. To investigate the relationship between internal training load and wellness, physiological, isokinetic profiling, and technical performance, the Pearson correlation coefficient (r) and coefficient of determination (r^2^) were used. The magnitude of associations was reported as follows: trivial r < 0.1, small 0.1 < r < 0.3, moderate 0.3 < r < 0.5, large 0.5 < r < 0.7, very large 0.7 < r <0.9, 0.9 < r < 1.0, nearly perfect [26]. The alpha level for the evaluation of statistical significance was set at *p* < 0.05.

## 3. Results

**Figure 2:**
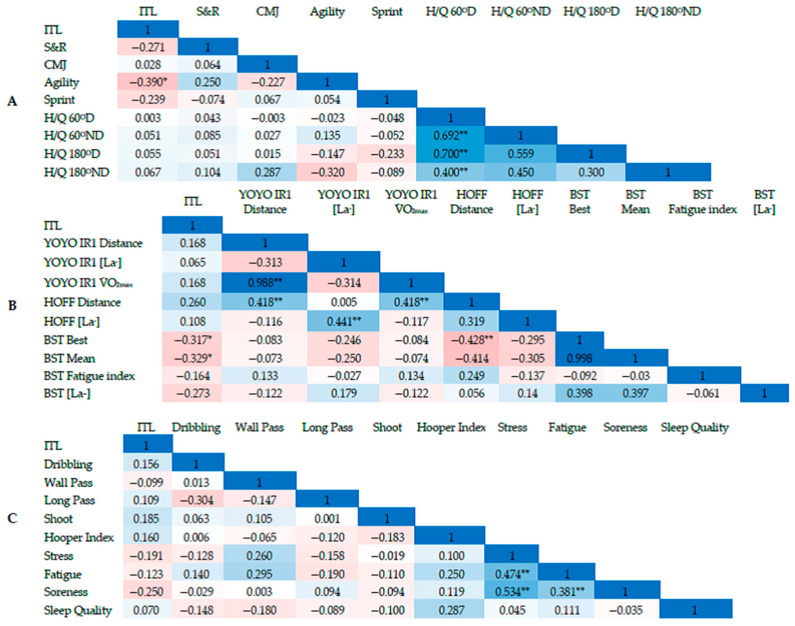
The Pearson correlation between the internal training load and (**A**) physical performance and isokinetic tests, (**B**) soccer-specific fitness tests, and (**C**) Soccer-specific technical skills and wellness status. The correlation magnitude of associations was reported as: trivial if r ≤ 0.1, small if r = 0.1 to 0.3, moderate if r = 0.3 to 0.5, large if r = 0.5 to 0.7, very large if r = 0.7 to 0.9, and nearly perfect if r ≥ 0.9. (** Correlation is significant at the 0.01 level; * Correlation is significant at the 0.05 level.) Legend: Blue color: direct correlation; Red color: inverse correlation; ITL: Internal training load; S&R: Sit and reach; CMJ: Counter-movement jump; Agility: Illinois agility test; Sprint: 30 m Sprint; H/Q 60°D: H/Q dominant ratios 60°·s^−s^; H/Q 60°ND: H/Q non-dominant ratios 60°·s^−s^; H/Q 180°D: H/Q dominant ratios 180°·s^−s^; H/Q 180°ND: H/Q non-dominant ratios 180°·s^−s^); YOYO IR1 Distance: YOYO IR1 distance; YOYO IR1 [La^−^]: YOYOIR1 Peak blood lactate; YOYO IR1, $\dot{V}$O_2max_: YOYO IR1 maximal oxygen uptake; HOFF Distance: Hoff test total distance; HOFF [La^−^]: Hoff test peak blood lactate; BST Best: Fastest Bangbo sprint test; BST Mean: Average Bangbo sprint test; BST Fatigue Index: Fatigue index of Bangbo sprint test; BST [La^−^]: Peak blood lactate of Bangbo sprint test); Dribbling: Slalom dribbling; Shoot: Shooting; Soreness: Muscle soreness; Sleep Quality: Sleep quality.

Descriptive statistics of internal training load (ITL), Hooper index (HI), and wellness status of young soccer players through the six-week pre-season structured combined training program are presented in Table 1. Pearson parametric correlations between internal training load and wellness status, physiological and biomarker responses, isokinetic profiling, and specific soccer skills are presented in Figure 2. The most remarkable result to emerge from the data are those significant correlations between internal training load had a moderate negative correlation with Illinois agility (r = −0.39, *p* = 0.01), BST best sprint (r = −0.32, *p* = 0.04), and BST mean sprint (r = −0.33, *p* = 0.03), respectively. Considering wellness status, moderate correlations were observed between average fatigue and average stress (r = 0.47, *p* < 0.01) and average muscle soreness (r = 0.38, *p* = 0.01), while a large correlation was observed between average stress and muscle soreness (r = 0.53, *p* < 0.01). However, there were no significant correlations between the wellness index and physiological, isokinetic, or specific soccer skill parameters.

Regarding the isokinetic profiling and soccer-specific fitness, results revealed moderate to very large correlations of H/Q ratios of dominant leg at the velocity of 60°·s^−1^ and non-dominant leg at the velocity of 180°·s^−1^ (r = 0.40, *p* < 0.01), non-dominant leg at the velocity of 60°·s^−1^ (r = 0.69, *p* < 0.01), and dominant leg at the velocity of 180°·s^−1^ (r = 0.70, *p* < 0.01). There was a significant positive correlation between Hoff-test and YO-YO IR1 (r = 0.42, *p* = 0.01), Hoff-test and $\dot{V}$O_2max_ (r = 0.42, *p* = 0.01), as well as YO-YO IR1 peak [La^-^] and Hoff peak [La^−^] (r = 0.44). A moderate negative correlation was found between the Hoff-test and BST best sprint (r = −0.43, *p* = 0.01). There was a statistically significant, moderately positive correlation between physical performance and soccer-specific fitness between the 30 m sprint and BST fatigue index (r = 0.41).

Significant improvements in 30 m sprint performance (*p* < 0.01; d = 0.90; −3.41%) were obtained in the physical performance variables. Interestingly, the soccer-specific fitness variables were all significantly improved (*p* < 0.01; d = 0.43 to 1.45; 4.85 to 60.97%) after the six-week pre-season training program. The difference in H/Q ratios at POST was higher than PRE condition at the velocity of 60°·s^−1^ in both dominant (*p* = 0.01; d = 0.17; 1.54%) and non-dominant leg (*p* = 0.02; d = 0.22; 2.96%) and at the velocity of 180°·s^−1^ in dominant leg (*p* < 0.01; d = 0.22; 2.78%). The four soccer-specific skills were all significantly improved (*p* = 0.01 to 0.04; d = 0.08 to 0.34; 0.64 to 9.45%), and the highest percentage of improvement was found in long pass (9.45%) (Table 2).

## 4. Discussion

The purpose of this study was to investigate whether a relationship existed between internal training load and wellness, physiological, isokinetic profiling, and technical performance. Furthermore, it evaluated changes and improvements in soccer-specific fitness, isokinetic profiling, and specific skills before and after a six-week pre-season SCPF, SSG, and SBT program in young national-level soccer players. Overall, there were no significant correlations between the wellness index and physiological, isokinetic, or specific soccer skill parameters. Moderate correlations between average fatigue and average stress, average muscle soreness, and large correlations between average stress and muscle soreness were presented during the pre-season training. Interestingly, regarding the improvement during the six weeks of the pre-season phase, young national soccer players improved soccer-specific physical fitness domains, with the BST fatigue index showing the greatest improvement, followed by YO-YO IR1 performance and YO-YO peak [La^−^], respectively. These findings support our hypothesis, and as a result, an organized multicomponent training program with the potential for physiological and technical adaptation and readiness, as well as managing structural training and monitoring stress responses, could induce suitable adaptation and balance psychological and physical well-being during the pre-season. Specifically, our results may suggest that a suitable structural training program incorporating monitoring of training load will also be important to the development of physical and technical skills, optimize the young national-level soccer player, and diminish the unbalance in athletic well-being [17].

Considering training load, the average total weekly training load in the present study (286.6) was lower than the previous study conducted in elite professional soccer (308.3) [15], with a systematic increasing pattern of loading being observed (272.67 to 298.24). A potential explanation for these findings is that the analysis does not consider various training patterns, performance levels, and players’ positions and roles, all of which might potentially impact the quantity of training loads [27]. The correlation analysis revealed a moderately negative correlation between average weekly internal training load, agility, and repeated sprint performance. Our results have a number of similarities with Buchheit et al. [28] in the sense that pre-season training focusing on physical abilities and technical and tactical skills can directly improve repeated-sprint running and aerobic performance. Interestingly, the Hooper index did not directly influence any other physical capacities or technical skills during pre-season training. It appears that the structured combined training program had no negative impact on well-being status adaptability and homeostasis restoration since there was less variability in different wellness indexes [11,16]. Furthermore, these findings are consistent with previous research that indicated significant interactions among wellness factors [16] have been identified during the training season, and the association between well-being status was particularly low in the pre-season period [11].

The current study demonstrated that the 30 m sprint exhibited the most significant enhancements when comparing the results following a six-week pre-season. Together, these findings indicate that enhancing neuromuscular components, specifically the hamstring to quadriceps strength ratio (H/Q ratio), could have a significant role in improving lower limb strength after training [8,9,29]. These findings corroborate previous studies that confirmed that eight weeks of biweekly combined plyometric and short sprint training [8] and plyometric and directional training have potentiated development in speed and jump performance in elite young soccer players [29]. However, our results showed significantly lower values for 30 m sprint improvement (−3.41%) than those reported in U-19 soccer Players (−9.00%) [8]. This is not surprising considering the pre-season period, which reinforces the efficacy of such a training strategy that primarily focuses on improving aerobic capacity and general strength.

Following the six-week preseason training program, there were significant improvements in all selected measurements related to soccer-specific fitness. Significant improvements were observed in all soccer-specific fitness variables (ranging from 3.54% to 60.97%; *p* < 0.001) from the beginning to the end of the pre-season training. The most significant enhancements were observed in the BST fatigue index (−60.97%), followed by YO-YO IR1 performance (25.31%) and YO-YO peak [La^−^] (19.82%), respectively. Our results support previous findings and provide further evidence on the importance of pre-season training, which aims to improve general aerobic endurance fitness, both physical and biological, to maximize metabolic and biomarker adaptations and the transition to the competitive phase, particularly in young soccer players [6,30].

In the present study, the YO-YO IR1 performance of young national soccer players (2218.09 ± 434.87 m) was slightly lower than that shown in previous studies on U-14 soccer players after the 12-week pre-season phase (2761 ± 716 m) [18] and U-17 and U-19 high-level soccer players (2404 ± 347 m, 2547 ± 337 m, respectively) [16]. On the other hand, results were slightly higher than data previously obtained in U-15 high-level soccer players (2024 ± 470 m) [21].

Furthermore, the current study revealed a moderate effect improvement in $\dot{V}$O_2max_ (4.85%; 55.03 + 2.80 mL·min·kg^−1^), mean BST time (−11.82%), and a small effect improvement in Hoff dribbling distance (3.45%) after the six-week pre-season phase. The improvement in aerobic fitness ($\dot{V}$O_2max_) in our study is lower than those shown in previous studies in young soccer players after 10 weeks of high-intensity aerobic interval training (63.4 to 69.8 mL·min·kg^−1^) [31]. Notwithstanding, our finding is consistent with the results in young elite Czech soccer players after 10 weeks of pre-season (55.6 to 57.2 mL·min·kg^−1^) [32]. The rate of $\dot{V}$O_2max_ improvement (4.85%) is slightly lower than expected, and there is certainly room for improvement as well as possible explanations for this result. The limitation of a structured combined training period consisting of four weeks of basic endurance capacity development followed by two weeks of speed endurance is very likely to have resulted in only trivial increases in $\dot{V}$O_2max_.

In fact, soccer performance has intermittent characteristics that require a high level of aerobic energy production and anaerobic capacity [13]. Previous research on the physiological determinants of aerobic performance has primarily focused on aerobic power ($\dot{V}$O_2max_) [33,34] and biochemical markers related to anaerobic metabolism and muscular acidosis (blood lactate concentration: [La^−^]) [33], from the YO-YO IR1 test, and from the Hoff test [20]. For young soccer’s anaerobic performance, the BST variables, including fatigue index and [La^−^], have been used to monitor and prescribe fatigue in soccer [34]. Peak [La^−^] levels 1–5 min after the aerobic exercise test observed in this study decreased from baseline to the end of the training period for the YO-YO IR1 test (7.98 ± 2.27 vs. 6.66 ± 1.84 mmol·L^−1^), as well as for the Hoff test (6.47 ± 1.77 vs. 5.50 ± 1.68 mmol·L^−1^). Our study confirmed previous findings [20,33,34] that pre-season training improved maximal aerobic capacity, distance covered, and the ability to sustain blood lactate concentrations during high-intensity intermittent running, as evaluated by the YO-YO IR1 test and the Hoff test. Furthermore, the significant contribution and improvement of the aerobic system would result in improved anaerobic endurance, which could directly contribute to improved match performance [34]. For anaerobic performance, the BST peak [La^−^] increased after the intervention period (7.68 ± 1.78 vs. 8.49 ± 1.99 mmol·L^−1^). Peak [La^−^] concentrations increased after the pre-season period, which could be attributed to increased buffering capacity and muscle adaptations [35], as a result of the main objectives of the training program, which was designed based on a structured combined training program for systematic and comprehensive improvement.

According to the results, it appears that a strong positive relationship existed among the Hoff test distance and both YO-YO IR1 and $\dot{V}$O_2max_. A negative correlation was additionally observed between the Hoff test distance and the BST best sprint. Consequently, the Hoff test performance was significantly correlated with other specific soccer measured aerobic fitness throughout the improvement in YO-YO IR1 and $\dot{V}$O_2max_ and the BST, which were reflected, particularly, in improved aerobic performance and repeated ability in young soccer players. The results of our study are in line with the findings of Pasquarelli et al. [19] and Chamari et al. [20], as the authors support the idea that soccer-specific testing can be extremely beneficial in terms of providing novel and insightful information to individual training plans and monitoring training intensities for the management of developing a periodization plan, particularly during pre-season periods [36]. The BST fatigue index, expressed as a percentage, which quantifies the prevalence of anaerobic effort, showed a notable enhancement (60.97%) subsequent to the training program. In addition, a substantially negative connection (r = −0.41) was seen between the BST fatigue index and the 30-m sprint. The results of our study line up with those of Pasquarelli et al. [19], who found a statistically significant association between the BST fatigue index and 10 to 30-m sprinting performance.

The national-level young male soccer players generally showed a trivially improved conventional H/Q ratio at slow testing velocity (60°·s^−1^) in both dominant (0.64 vs. 0.65) and non-dominant (0.65 vs. 0.67) while the H/Q ratio of dominant leg was only significantly improved at high testing velocity (180°·s^−1^) (0.70 vs. 0.72). In addition, there was no significant difference in H/Q ratios between the dominant and non-dominant at slow testing velocity (60°·s^−1^) to indicate maximal peak torque and at high testing velocity (180°·s^−1^) to simulate functional athletic movements after a six-week training period. The results of our study align with the previous study, which stated that the normative H/Q ratio was approximately 0.61 at 60°·s^−1^ and 0.72 at 180°·s^−1^ [37] and the mean value was between 51–80% at intermediate velocities [38]. Furthermore, our findings agree with previous studies that showed no notable difference between the dominant and non-dominant legs in professional soccer players [39]. The present study highlights that the conventional H/Q ratio was greater at a velocity of 180°·s^−1^ in comparison to 60°·s^−1^. These findings indicate that young soccer players displayed the greatest H:Q strength ratio and improved knee stabilization at a high testing velocity (180°·s^−1^). Consequently, it is reasonable to conclude that incorporating resistance, strength, and conditioning training during the pre-season stage into the training of young soccer players may enhance performance. Such training can serve as a stimulus for hormonal and metabolic adaptations, specifically affecting growth hormone and testosterone levels, particularly in the age groups U-16 and U-18 [38,40].

The technical skills of dribbling slalom, wall pass, long pass, and shooting showed substantial improvements following the pre-season period. From a perspective of mastery technical skills, young elite technical skills depend on a series of important factors, such as biological maturity status, players’ position, and physical fitness [3,30]. Furthermore, some evidence suggests that physical fitness improvement has an impact on the performance of soccer-specific skills, particularly dribbling speed [4], and thus plays an important role in young soccer performance [30], particularly in the pre-season training period. Even though technical skills and physical fitness are important in pre-season, no relationship was found between physical performance, isokinetic strength, soccer-specific fitness, and soccer-specific skills. The lack of correlation between soccer-specific skills and the variables selected may be attributed to the fact that young soccer players were grouped together for evaluation without considering the variations in their positions and roles. Consequently, it is important to include an examination of soccer playing positions in future research, especially when studying samples of young soccer players who have reached puberty [27,30].

Finally, future research should consider a number of potential limitations that may have influenced the results obtained. Firstly, because the current study used a one-group pre-test-post design with a short (six-week) time interval and variation by playing position was not considered, the improvement of young soccer performance and the role of the relationships between physical performance, soccer-specific fitness and biomarker adaptations, isokinetic strength, and soccer-specific motor skills should be interpreted with caution. Secondly, it should be noted that the results of the current study are only appropriate for young national soccer players’ levels and should not be generalized to other soccer player performance levels. This means that the soccer players who have different performance levels from the national young soccer players in the current study, such as young elite and professional soccer players, might show different results. Finally, yet importantly, a control group should be added to future studies, allowing for the minimization of random effects on dependent variables over time and obtaining stronger experimental research designs.

## 5. Conclusions

This study aimed to quantify the relationships among internal training load, wellness, physiological profile, isokinetic profile, and technical performance. It assessed changes before and after a six-week pre-season structured program combining physical fitness, small-sided games, and skill-based training in young soccer players. Overall, no significant correlations were found between the wellness index and physiological, isokinetic, or specific soccer skill parameters. Moderate correlations were observed between fatigue, stress, and muscle soreness, while a large correlation was found between average stress and muscle soreness. Additionally, improvements in physiological, isokinetic profile, and technical parameters were noted after the six-week pre-season training program. Therefore, it is crucial to focus on effectively managing and monitoring the training process to provide pertinent information to coaches and strength and conditioning trainers. This information can be directly applied to developing strategies for managing the development of young soccer players. A practical approach for coaches is to implement systematic training methods that emphasize enhancing physical fitness levels, including repeated sprint running, aerobic performance, and lower limb strength during preseason training. Such an approach has proven beneficial, especially for young players. Moreover, conducting comprehensive physical assessments and adjusting the intensity of internal training and well-being indicators for young soccer players at the beginning of the season can provide coaches with a foundation for improving periodization and reducing the risk of stress-related injuries. Finally, to effectively enhance players’ physical well-being and psychological performance, it is essential to harmonize internal training intensity and well-being status.

## Figures and Tables

**Table 1:** Descriptive statistics (M ± SD) of internal training load (ITL), Hooper index (HI), and each wellness status of young soccer players through the six-week pre-season structured combined training program. AU: arbitrary units.

VARIABLES	WEEK 1	WEEK 2	WEEK 3	WEEK 4	WEEK 5	WEEK 6	mMEAN ± SD
**Internal training load (A.U.)**	272.67 ± 8.25	278.50 ± 7.21	284.31 ± 5.89	290.07 ± 4.18	294.10 ± 6.21	298.24 ± 6.20	286.64 ± 3.96
**Hooper index (HI)**	11.82 ± 0.55	11.90 ± 0.55	12.32 ± 0.41	12.62 ± 0.49	12.71 ± 0.49	13.14 ± 0.48	12.42 ± 0.22
Stress	3.02 ± 0.23	3.05 ± 0.19	3.15 ± 0.20	3.25 ± 0.21	3.35 ± 0.22	3.36 ± 0.20	3.20 ± 0.14
Fatigue	2.91 ± 0.26	2.95 ± 0.22	3.00 ± 0.26	3.11 ± 0.27	3.17 ± 0.30	3.25 ± 0.28	3.07 ± 0.19
Muscle Soreness	3.03 ± 0.22	3.03 ± 0.19	3.13 ± 0.14	3.23 ± 0.15	3.23 ± 0.14	3.43 ± 0.33	3.18 ± 0.12
Sleep Quality	3.41 ± 0.21	3.30 ± 0.21	3.21 ± 0.20	3.22 ± 0.16	3.12 ± 0.13	3.10 ± 0.16	3.23 ± 0.11

**Table 2:** Effects of a six-week pre-season structured combined training program on physical performance, soccer-specific fitness tests and biomarker responses, specific soccer skills, and the hamstring-to-quadriceps strength ratio (H/Q ratio) improvement. Data are displayed as the PRE and POST mean ± SD values, 95% confidence intervals, comparison *p*-value, and effect size.

VARIABLES	PRE	POST	Diff. PRE-POST, Δ%	*t*-Test	*p*-Value	Cohen’s d
**PHYSICAL PERFORMANCE**						
**Sit and reach** (cm)	12.41 ± 6.72	12.97 ± 7.04	0.50; 4.51%	−4.05	=0.11	0.15, Trivial
**Illinois agility** (s)	15.81 ± 0.55	15.71 ± 0.61	−0.09; −0.64%	1.33	=0.19	0.17, Trivial
**CMJ** (cm)	38.70 ± 6.33	38.22 ± 3.92	−0.48; −1.26%	0.58	=0.57	0.09, Trivial
**30 m sprint** (s)	4.24 ± 0.17	4.10 ± 0.16	−0.14; −3.41%	4.98	<0.01	0.85, Moderate
**SOCCER SPECIFIC FITNESS TESTS**						
**YO-YO IR1** (m)	1656.67 ± 333.61	2218.09 ± 434.87	561.83; 25.31%	−10.97	<0.01	1.45, Large
$\dot{V}$**O_2max_** (mL·min^−1^·kg^−1^)	52.36 ± 3.65	55.03 ± 2.80	2.72; 4.85%	−6.32	<0.01	0.83, Moderate
**YO-YO peak [La^−^]** (mmol·L^−1^)	7.98 ± 2.27	6.66 ±1.84	−1.32; −19.82%	9.31	<0.01	0.64, Moderate
**Hoff-test** (m)	1515.24 ± 148.97	1569.45 ± 146.47	54.21; 3.45%	−3.93	<0.01	0.37, Small
**Hoff-peak [La^−^]** (mmol·L^−1^)	6.47 ± 1.77	5.50 ± 1.68	−0.97; −17.64%	5.67	<0.01	0.56, Small
**BST best sprint** (s)	6.97 ± 1.30	6.37 ± 0.83	−0.59; −9.42%	4.55	<0.01	0.55, Small
**BST mean time** (s)	7.38 ± 1.37	6.60 ± 0.86	−0.76; −11.82%	5.63	<0.01	0.68, Moderate
**BST fatigue index** (%)	5.65 ± 1.53	3.51 ± 0.86	−2.14; −60.97%	7.58	<0.01	1.81, Large
**BST peak [La^−^]** (mmol·L^−1^)	7.68 ± 1.78	8.49 ± 1.99	0.81; 9.54%	−8.23	<0.01	0.43, Small
**ISOKINETIC TEST**						
**H/Q dominant ratios 60°·s^−1^**	0.64 ± 0.06	0.65 ± 0.06	0.02; 1.54%	−2.59	=0.01	0.17, Trivial
**H/Q non-dominant ratios 60** **°·s^−1^**	0.65 ± 0.09	0.67 ± 0.09	0.02; 2.96%	−2.52	=0.02	0.22, Small
**H/Q dominant ratios 180°·s^−1^**	0.70 ± 0.09	0.72 ± 0.09	0.02; 2.78%	−3.35	<0.01	0.22, Small
**H/Q non-dominant ratios 180** **°·s^−1^**	0.73 ± 0.08	0.73 ± 0.10	0.00; 0.10%	−0.07	=0.94	0.09, Trivial
**SOCCER SPECIFIC SKILLS**						
**Dribbling slalom** (s)	17.17 ± 1.38	17.06 ± 1.42	−0.10; −0.64%	3.02	<0.01	0.08, Trivial
**Wall pass 20 s** (n)	17.55 ± 2.98	17.88 ± 2.54	0.33; 1.86%	−2.86	<0.01	0.12, Trivial
**Long pass** (points)	15.33 ± 3.73	16.93 ± 2.49	1.60; 9.45%	−2.07	=0.04	0.12, Trivial
**Shooting** (points)	17.83 ± 1.41	18.24 ± 0.93	0.40; 2.48%	−2.59	<0.01	0.34, Small

## Data Availability

The data presented in this study are only available upon request from the corresponding author. The data are not publicly available due to their containing of information that could compromise the privacy of study participants.
